# Coverage of the 2011 Q Fever Vaccination Campaign in the Netherlands, Using Retrospective Population-Based Prevalence Estimation of Cardiovascular Risk-Conditions for Chronic Q Fever

**DOI:** 10.1371/journal.pone.0123570

**Published:** 2015-04-24

**Authors:** Patricia E. Vermeer-de Bondt, Teske Schoffelen, Ann M. Vanrolleghem, Leslie D. Isken, Marcel van Deuren, Miriam C. J. M. Sturkenboom, Aura Timen

**Affiliations:** 1 Centre for Infectious Disease Control, National Institute for Public Health and Environment, Bilthoven, the Netherlands; 2 Department of Internal Medicine, Radboud University Medical Center, Nijmegen, the Netherlands; 3 Department of Medical Informatics, Erasmus University Medical Center, Rotterdam, the Netherlands; Texas A&M Health Science Center, UNITED STATES

## Abstract

**Background:**

In 2011, a unique Q fever vaccination campaign targeted people at risk for chronic Q fever in the southeast of the Netherlands. General practitioners referred patients with defined cardiovascular risk-conditions (age >15 years). Prevalence rates of those risk-conditions were lacking, standing in the way of adequate planning and coverage estimation. We aimed to obtain prevalence rates retrospectively in order to estimate coverage of the Q fever vaccination campaign.

**Methods:**

With broad search terms for these predefined risk-conditions, we extracted patient-records from a large longitudinal general-practice research-database in the Netherlands (IPCI-database). After validation of these records, obtained prevalence rates (stratified for age and sex) extrapolated to the Q fever high-incidence area population, gave an approximation of the size of the targeted patient-group. Coverage calculation addressed people actually screened by a pre-vaccination Q fever skin test and serology (coverage) and patients referred by their general practitioners (adjusted-coverage) in the 2011 campaign.

**Results:**

Our prevalence estimate of any risk-condition was 3.1% (lower-upper limits 2.9-3.3%). For heart valve defects, aorta aneurysm/prosthesis, congenital anomalies and endocarditis, prevalence was 2.4%, 0.6%, 0.4% and 0.1%, respectively. Estimated number of eligible people in the Q fever high-incidence area was 11,724 (10,965-12,532). With 1330 people screened for vaccination, coverage of the vaccination campaign was 11%. For referred people, the adjusted coverage was 18%. Coverage was lowest among the very-old and highest for people aged 50–70 years.

**Conclusion:**

The estimated coverage of the vaccination campaign was limited. This should be interpreted in the light of the complexity of this target-group with much co-morbidity, and of the vaccine that required invasive pre-vaccination screening. Calculation of prevalence rates of risk-conditions based on the IPCI-database was feasible. This procedure proved an efficient tool for future use, when prevalence estimates for policy, implementation or surveillance of subgroup-vaccination or other health-care interventions are needed.

## Introduction

**Fig 1:**
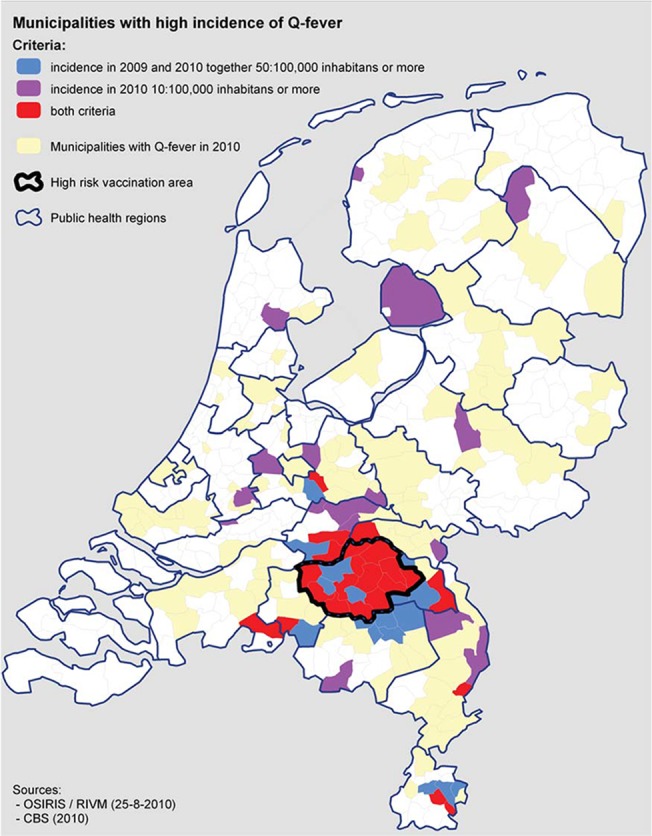
Municipalities with their incidence of Q fever in 2009–2010. Q fever high-incidence area outlined in black (11).

In 2007, a large human Q fever outbreak started in the southeast of the Netherlands, caused by exposure to goat-farms infected by *Coxiella burnetii* [1]. Subsequently, the annual number of reported human Q fever cases reached a peak of 2354 in 2009. The increase of acute Q fever reports was followed by increasing numbers of chronic Q fever patients [2]. Chronic Q fever is a severe outcome of *C*. *burnetii* infection, often presenting itself as endocarditis or vascular infection, and needs long-term antibiotic treatment and often surgical intervention [3]. Several cardiovascular risk-conditions for chronic Q fever have been identified [4,5], and the Health Council (HC) of the Netherlands advised vaccination of people aged 15 years and over, with these specific risk-conditions in July 2010 [6]. This measure was an individual patient-oriented intervention and not a population-targeted campaign. The sole available vaccine, only registered in Australia where it is used to vaccinate people at occupational risk [7,8], requires pre-vaccination screening [9,10], and therefore complicated logistics [11]. The Ministry of Health chose a centralized approach, coordinated by the National Institute for Public Health and Environment. Efforts to reach the target population concentrated on high-incidence areas [Fig 1] and relied on general practitioners (GPs), and to a lesser extent to hospital specialists, for patient referrals. Intake, screening and subsequent vaccination was organized at the municipal health clinic in the central high-incidence area. The vaccination campaign included intensified safety surveillance [12], immunological pre-vaccination screening and immunological follow-up [11,13].

In their advice, the HC did not attempt to quantify the size of the risk-group eligible for vaccination and no concise prevalence data of the risk-conditions were available according to cardiologists. In the planning phase of the vaccination campaign, which should start before the approaching lambing season, it was estimated that 100–300 people would respond to the invitation for vaccination within the target population, and 1000 was taken as upper limit budget wise. The much larger than expected number of referred people (2741) overloaded the system and made implementation of the campaign difficult, forcing an up-scaling of staff [11]. In the end, 1786 eligible people underwent screening tests between January and April 2011.

The lack of prevalence data of the risk-conditions not only precluded determination of the size of the target population, but also of the coverage of the vaccination campaign. In order to estimate coverage retrospectively, we needed to obtain prevalence estimates of the risk-conditions for chronic Q fever in the high-incidence area. To this end, we extracted data on the specific risk-conditions from the Integrated Primary Care Information (IPCI)-database, a large longitudinal population-based general practice research database in the Netherlands. Here we describe the procedure we followed to arrive at an estimate of the coverage of the Q fever vaccination campaign in the Netherlands 2011 [14].

## Materials and Methods

### IPCI-database

The IPCI-database is a longitudinal general practice research database held by Erasmus University Medical Center, Rotterdam. With data collection since 1996, this electronic dynamic database presently contains 1.1 million patient-records from 437 general practices in the Netherlands [15–17]. It holds comprehensive information on medical history and referrals. Included are signs and symptoms, diagnoses, International Classification of Primary Care (ICPC)-codes, laboratory test results, surgical procedures, medication and hospitalizations. The free text fields of the records contain summaries or full discharge letters from hospital specialists. Hard copies of letters are available upon request. The patient population is representative of the Dutch population, with some underrepresentation of elderly people who have moved to nursing homes however [17]. Patient-records are anonymous for researchers. IPCI complies with European Union guidelines on secondary use of healthcare data for medical research. The IPCI-database is valid for pharmaco-epidemiological research and has been used in more than 100 peer-reviewed publications. The Supervisory Board of the IPCI-database (project number 11/2012) approved the use of IPCI-data for the current prevalence estimation. Approval by the ethics committee was not necessary for this retrospective study because only anonymous data have been used.

### Study population

The source population comprised all patients 15 years and older who were registered with one of the GP-practices and actively in follow-up with the GP on January 1^st^ 2011 [6]. Due to resource limitations regarding the necessary manual validation of extracted patient-records, we sampled 5% of this source population as study population. We compared the characteristics of the full source population and those of the 5% random sample for consistency in pattern of non-validated potential cases.

### Risk-conditions and search criteria

Four risk-conditions were defined, based on the HC-advice of eligibility for Q fever vaccination [6]. Relevant search terms were entered in a script (syntax) with which the database was searched in diagnostic code sections and free text fields [Table 1]. Physicians (PEVdB and TS) reviewed the electronic medical records of all potential cases in order to assess validity and label the diagnostic certainty. They entered the level of diagnostic certainty (definite-probable-possible case or no case) in a validation tool with date of first diagnosis or of rejection. Cases could have multiple risk-conditions. We did not subdivide the cases according to severity or type of defect within the specific risk-conditions, since this was not the scope of our study. A random sample of 100 records was crosschecked by independent review (AMV). These records and all cases with the lowest level of diagnostic certainty (possible) were thereafter checked for consistency, held against strict inclusion criteria, and coded following consensus (PEVdB and TS).

**Table 1:** Cardiovascular risk-conditions for chronic Q fever with ICPC-codes, search terms and diagnostic certainty levels.

Risk-conditions, ICPC-codes^a^, search terms and diagnostic certainty levels for IPCI-database^b^ extraction		
**Risk-conditions and ICPC-codes** ^**c**^	**1-**	valvular cardiac disease or prosthesis (symptomless mitral valve prolapse excluded) (K83)
	**2-**	aortic aneurysm of prosthesis/stent (K99)
	**3-**	congenital cardiac anomalies (except spontaneous closure of VSD/ASD/OBD or surgical closure without artificial material and no residual defect), inclusive of coarctatio aortae (K73)
	**4-**	history of endocarditis or rheumatic cardiac disease (K71)
**Search terms in free text**	**a-**	valv*, aort*, aneury*, congenit*, viti*, prosth*, vsd/asd, obd, mitra*, tricusp*, stenos*, insuff*, endocardi* and the Dutch equivalents, etcetera’s
	**b-**	proper names of different (congenital) syndromes like Eisenmenger, Fallot, Epstein, Ivemark, Botalli, etcetera’s. All these search terms included also possible spelling errors
**Level of diagnostic certainty**	**1-**	certain; with discharge letter of specialist or specific diagnostic laboratory outcome, or repetitive entries with description and specific treatment
	**2-**	probable; with only descriptions entered by the general practitioner, with the correct ICPC-codes
	**3-**	possible; with only (recurrent) ICPC-code, without description of condition, or minimal defects with uncertain consequences
	**4-**	rejected; if condition could be regarded as variant of normal, minimal defects without any consequences, or falling within exclusion criteria, as well as conditions excluded by specialist examination, or conditions in family members

^a^ ICPC-codes, International Classification of Primary Care codes

^b^ IPCI-database, Integrated Primary Care Information database

^c^ Qualifying patients were eligible for Q fever vaccination in the campaign of 2011

### Population of the Q fever high-incidence area and the Q fever vaccination campaign

The population distribution of the Q fever high-incidence area as of January 1^st^ 2011was retrieved from demographic data from the electronic databank Statline (Statistics Netherlands, http://statline.cbs.nl/Statweb). Information on actually referred and screened people in the Q fever vaccination campaign was obtained from the case report forms of the Q fever vaccination campaign as described previously [12]. Risk-conditions of referred people came from GPs and from the potential vaccinees themselves. Postal codes identified referred people living in the high-incidence area. Of 2741 potential vaccinees, 955 were excluded for various reasons [11]. The remaining 1786 people—of which 1330 from the high-incidence area—underwent pre-vaccination screening with skin test and serology, and—if negative—were subsequently vaccinated. Logistics and safety surveillance of the Q fever vaccination campaign have been described elsewhere [11,12].

### Data analysis

After validation and review of extracted patient-records from the IPCI-sample, stratified numbers on the four defined risk-conditions according to 10 years’ age groups and sex, were used to determine prevalence (N/10,000), with 95% Confidence Intervals (95%CI). We did so both for all confirmed cases and for cases with definite and probable diagnostic certainty only, with lower and upper limits.

**Fig 2:**
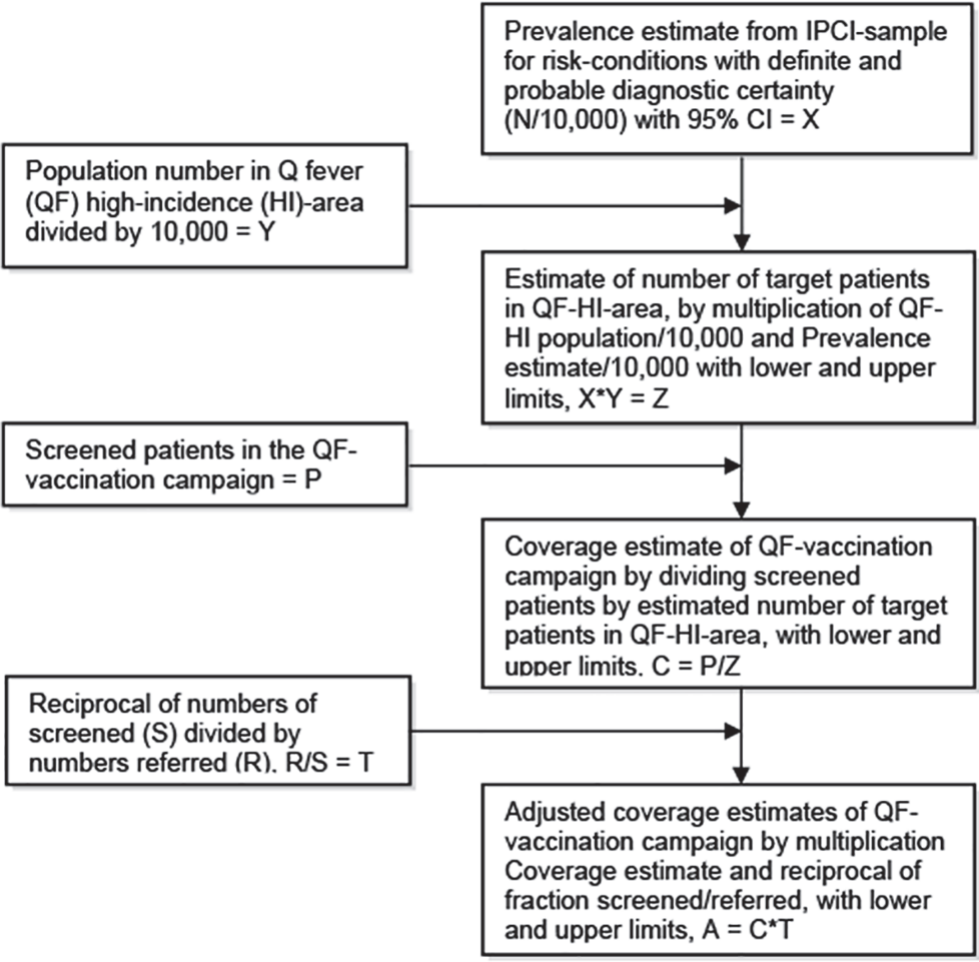
Flow diagram of calculations leading to coverage estimates. For calculations, prevalence of risk-conditions with definite and probable diagnostic certainty from IPCI-study population has been used, overall and for subgroups.

The prevalence estimates for cases with definite and probable diagnostic certainty, stratified for age group and sex, served as input for the high-incidence area population, to approximate the number of people eligible for Q fever vaccination in this region. With this, the coverage of the vaccination campaign was determined with minimum and maximum estimates based on the 95% CI of the IPCI-sample data. Fig 2 presents a diagram with the calculation steps. OpenEpi and Excel were used for calculations and graphs.

## Results

**Fig 3:**
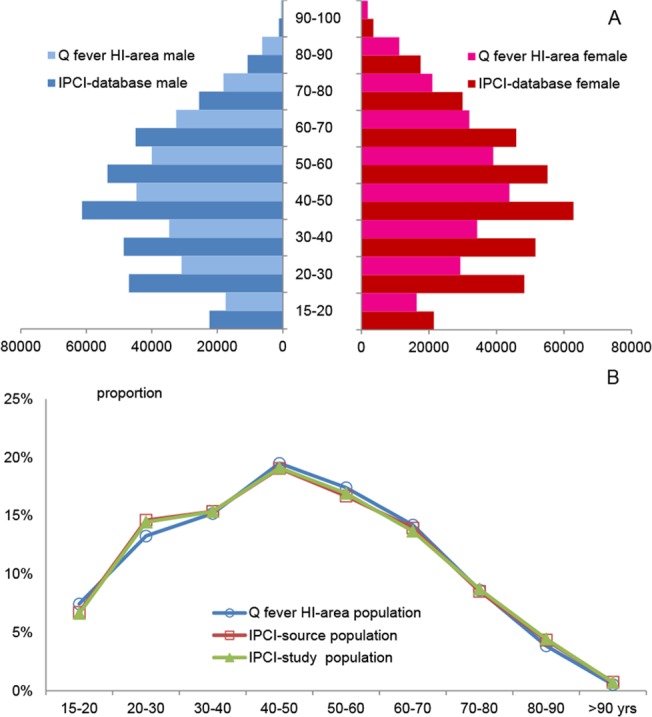
Population distribution of IPCI-database and of Q fever high-incidence area. (A) population pyramids for IPCI-database (dark colours) and for Q fever high-incidence (HI)- area (light colours), according to sex. (B) Relative age frequencies for IPCI-source population (open squares), IPCI-study population (triangles) and for Q fever HI-area (open circles).

The IPCI-database per January 1^st^ 2011 contained 651,276 people aged 15 years and over. The 5% random sample as study population had comparable age and sex distribution. The relative age distribution of the populations of the IPCI-sample and of the high-incidence area (as obtained from CBS) shows that they were similar (with equal median age), though the latter had slightly less people in the age group of 20–30 years and more in the 50–60 year olds [Fig 3].

**Fig 4:**
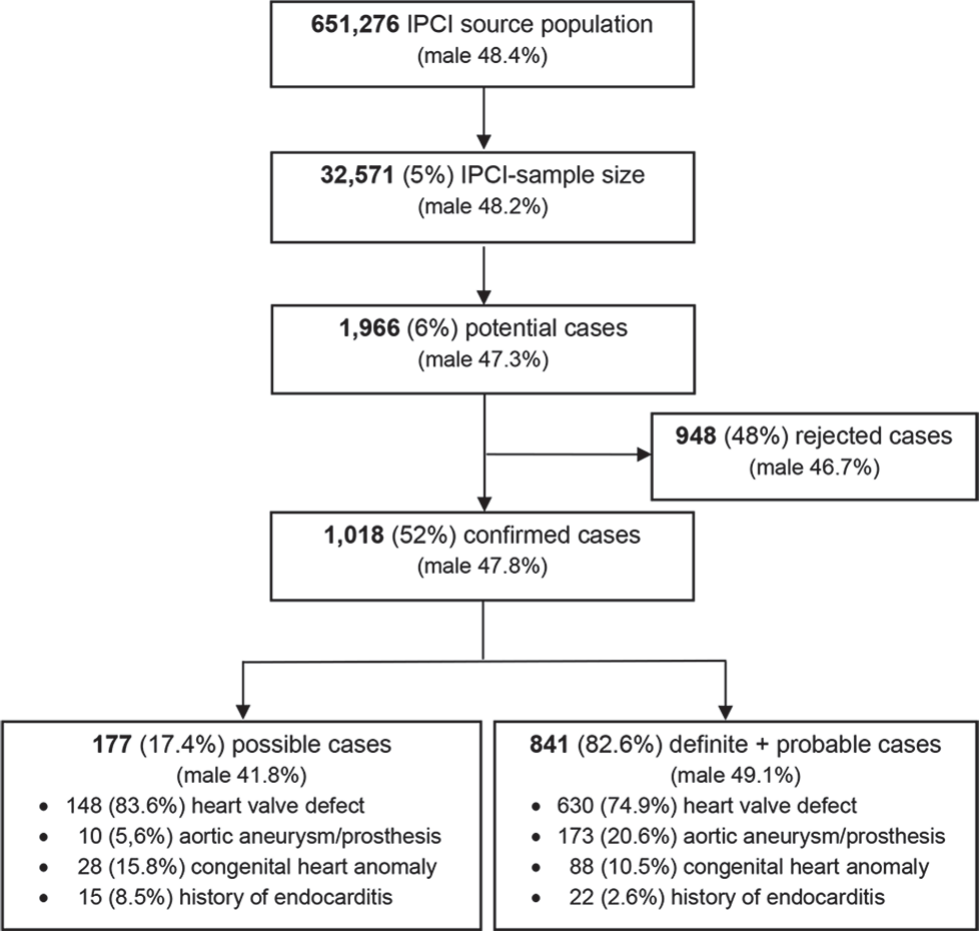
Flow diagram of results of IPCI-database extraction and validation. Sex distribution (male %) included for each step.

The IPCI-study population included 32,571 people [Table 2]. We identified 1966 patient-records (6%) with one or more ICPC-codes and/or search terms for chronic Q fever risk-conditions [Table 1], women (52.7%) slightly outnumbering men (47.3%). After review and validation, we rejected 948 extracted patients in all (viz. hits for family members with a risk-condition, or medical investigation ruling out a specified risk-condition, or non-qualifying diagnoses). This left 1018 (52%) cases with one or more confirmed chronic Q fever risk-conditions. Of these, 177 (17%) had possible diagnostic certainty and 841 definite or probable diagnostic certainty [Fig 4].

**Table 2:** Risk-conditions for chronic Q fever in the IPCI-study population, according to age groups and sex.

**IPCI-population, risk-conditions, prevalence rates and age groups**										
**IPCI-study population (number)**										
**Age group**	**15–20**	**20–30**	**30–40**	**40–50**	**50–60**	**60–70**	**70–80**	**80–90**	**>90 yrs**	**all**
**All**	2158	4708	5001	6224	5509	4454	2837	1447	233	32571
male	1093	2354	2400	3047	2728	2138	1300	579	72	15711
female	1065	2354	2601	3177	2781	2316	1537	868	161	16860
male%	51%	50%	48%	49%	50%	48%	46%	40%	31%	48%
**Risk-conditions (number)^a^**										
**Age group**	**15–20**	**20–30**	**30–40**	**40–50**	**50–60**	**60–70**	**70–80**	**80–90**	**>90 yrs**	**all**
**Heart valve defect**	3	9	14	54	67	166	225	199	41	778
**Aortic aneurysm/prosthesis**	1	2	0	7	19	38	63	49	4	183
**Congenital heart anomaly**	15	24	17	24	14	14	3	5	0	116
**Endocarditis**	0	1	3	4	7	12	6	4	0	37
**Any risk condition**	16	31	30	82	94	212	269	241	43	1018
male	6	16	11	34	44	111	142	110	13	487
female	10	15	19	48	50	101	127	131	30	531
male%	38%	52%	37%	41%	47%	52%	53%	46%	30%	48%
**Prevalence of risk-conditions (/10,000)^a^**										
**Age group**	**15–20**	**20–30**	**30–40**	**40–50**	**50–60**	**60–70**	**70–80**	**80–90**	**>90 yrs**	**all**
**Heart valve defect**	14	19	28	87	122	373	793	1375	1760	239
male	9	21	21	66	106	379	815	1347	1667	214
female	19	17	35	107	137	367	774	1394	1801	262
**Aortic aneurysm/prosthesis**	5	4	0	11	34	85	222	339	172	56
male	0	8	0	13	51	131	385	674	417	89
female	9	0	0	9	18	43	85	115	62	26
**Congenital heart anomaly**	70	51	34	39	25	31	11	35	0	36
male	55	51	29	39	22	33	15	17	0	34
female	85	51	38	38	29	30	7	46	0	37
**Endocarditis**	0	2	6	6	13	27	21	28	0	11
male	0	4	0	3	15	28	23	17	0	10
female	0	0	12	9	11	26	20	35	0	12
**Any risk-condition**	74	66	60	132	171	476	948	1666	1845	313
lower limit	46	46	42	106	140	417	846	1482	1400	294
upper limit	120	93	86	163	208	543	1062	1866	2393	332
male	55	68	46	112	161	519	1092	1900	1806	310
female	94	64	73	151	180	436	826	1509	1863	315

Distribution of the full IPCI-study population; number of confirmed risk-conditions in the IPCI-study population; prevalence estimates of risk-conditions per 10,000 according to sex, as calculated from the IPCI-study population, with lower and upper limits.

^a^ The scoring of risk-conditions as shown here, applies to all confirmed cases, including possible and probable/definite diagnostic certainty.

The majority of confirmed cases from the IPCI-study population (925/1,018; 91%) had only one defined risk-condition and 9.1% had multiple risk-conditions, mostly double and only three triple (male 11.5% and female 7.0%). Valvular disease was most prevalent (778; 148 possible), followed by aortic aneurysm or prosthesis (183; 10 possible), congenital cardiac anomalies (116; 28 possible) and endocarditis (37; 15 possible) [Table 2 and Fig 4].

**Fig 5:**
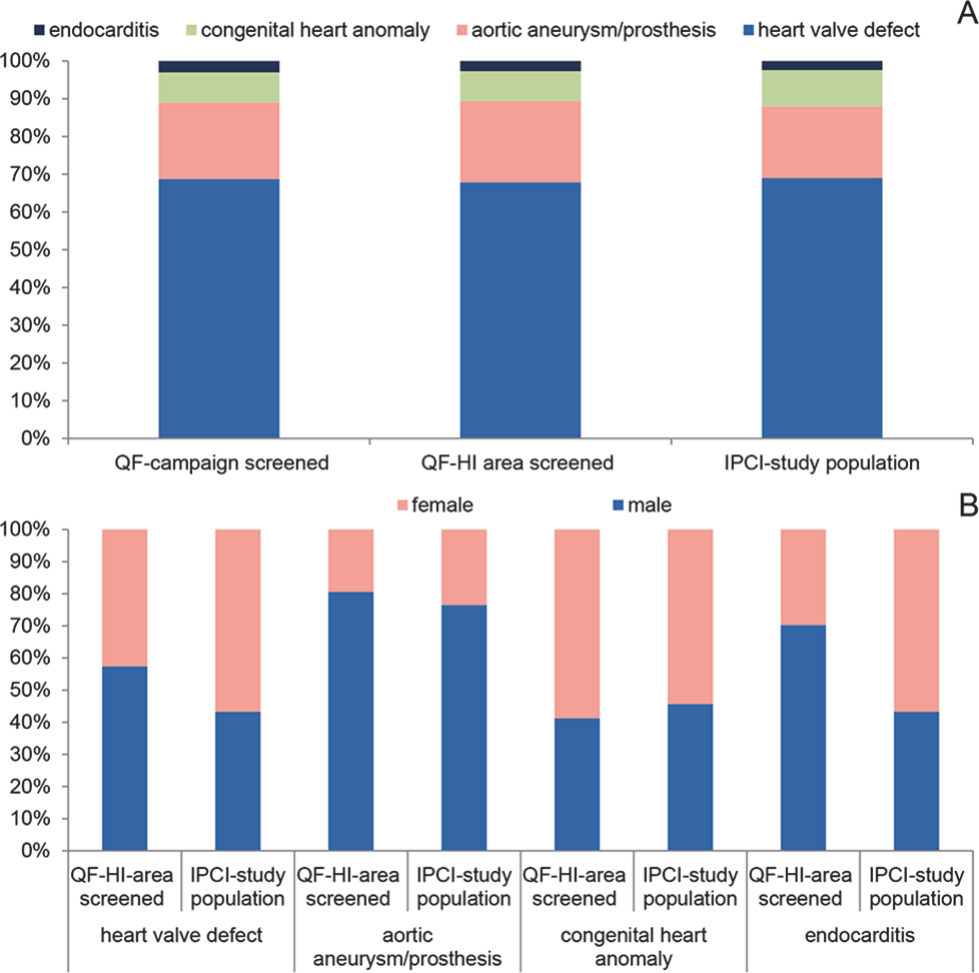
Relative frequencies of risk-conditions for chronic Q fever and sex distribution in the different populations. (A) Comparison between all screened people of the Q fever (QF)-vaccination campaign, screened people from the high-incidence (HI)-area, and cases from the IPCI-study population. (B) Sex distribution for different risk-conditions in screened people from QF-HI-area and cases from IPCI-study population. The IPCI-study population includes cases with definite and probable diagnostic certainty.

The relative frequencies of the four risk-conditions in the IPCI-study population were similar to those in the screened people of the Q fever campaign and the screened people from the high-incidence area [Fig 5A]. However, the screened people were younger than the confirmed cases with definite and probable diagnostic certainty from the IPCI-study population. Median (interquartile range) ages were 67 (57–74) and 72 (61–81) years for screened people and IPCI-cases, respectively. IPCI-cases with possible diagnostic certainty were younger and had a higher proportion of women (58% versus 51%). The proportion of men was higher in the referred and screened people of the Q fever vaccination campaign from the high-incidence area (61%) than in the IPCI-study population cases (48%), for all risk-conditions except for congenital heart anomalies [Figs 4 and 5B].

**Fig 6:**
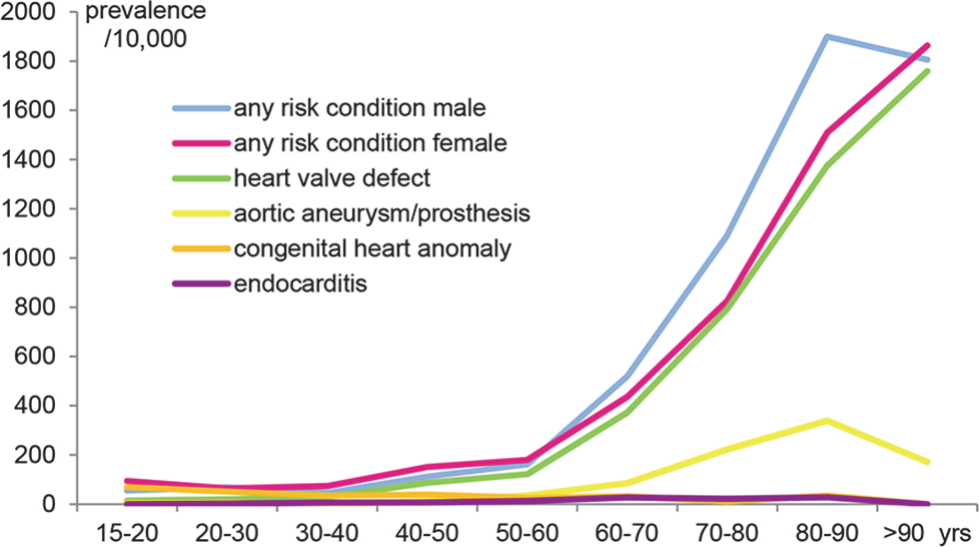
Age distribution of the defined risk conditions for chronic Q fever. Prevalence rates (per 10,000) are estimated from the IPCI-database, per January 1^st^ 2011.

The overall prevalence of any risk-condition in the IPCI-study population was 313 per 10,000 people, increasing with age [Fig 6]. Only for congenital anomalies, prevalence was highest in the youngest age group. Men and women showed almost equal overall prevalence rates of 310 and 315 per 10,000, respectively [Table 2]. These calculated prevalence rates for the different risk-conditions are within data ranges presented in literature [Table 3].

**Table 3:** Prevalence rates for separate cardiovascular risk conditions and comparison with literature.

Risk-conditions Prevalence/100	Overall	Range for age groups	Lower- upper limits	Male; peak	Female; peak	Range in literature	Lit ref #
**Heart valve defect**	2.39	0.1–17.6	2.23–2.56	2.14; 16.67	2.62; 18.01	0.2–13.0	28–33
**Aortic aneurysm/prosthesis**	0.56	<0.05–3.0	0.49–0.65	0.89; 6.74	0,26; 3.39	up to 8.9	33–38
**Congenital heart anomaly**	0.36	<0.1–0.7	0.30–0.43	0.34; 0.55	0.37; 0.85	0.4–0.8	39–44
**Endocarditis**	0.11	0–0.28	0.08–0.16	0.10; 0.28	0.12; 0.35	0.12	45–46
**Any risk condition**	3.1	0.6–18.5	2-94-3.32	3.10; 19.00	3.15; 18.63	-	-

Prevalence per 100 people with lower and upper limits and range over the age groups and sex distribution with peak prevalence.

To get an impression of the proportion of eligible people who were reached in the Q fever vaccination campaign (coverage), one should first calculate prevalence estimates for validated IPCI-cases with definite and probable diagnostic certainty [Fig 2]. These prevalence rates were stratified for age group and sex, with an overall rate of 258/10,000 [S1 Table]. Extrapolation of these rates to the population of the Q fever-high-incidence area, led to an estimated number of targeted people of 11,724 (10,965–12,532) [S2 Table]. If people from municipalities bordering the high-incidence area were included in this calculation, the target group would number at least 15,000 people.

Overall, only 1330 of this target group from the high incidence area were screened for vaccination [Table 4], which resulted in coverage of 11% (1330/11,724) with narrow lower and upper limits of 11–12%. For men and women these coverage rates were 14% (13%-15%) and 9% (8%-10%), respectively. For the larger group of referred people from the high-incidence area, an adjusted coverage rate of 18% (limits 16%-18%) was calculated. Coverage was lowest in the oldest age groups, with 5% and 1% for the 80–90 year olds and 90 years and older, respectively. The highest coverage was in the much larger groups of the 50–60 and 60–70 year olds, with 19% and 16% respectively (upper limits of 23% and 19%). Based on the referred people in these latter age groups, this would result in an adjusted coverage of approximately 30% and 24% (upper limits 34% and 29%) [Table 5].

**Table 4:** Risk-conditions for chronic Q fever of screened patients in the vaccination campaign in 2011.

Risk-conditions and age groups	15–20	20–30	30–40	40–50	50–60	60–70	70–80	80–90	>90 yrs	all
**Heart valve defect**	14	14	20	62	145	305	280	85	4	929
**Aortic aneurysm/prosthesis**	0	1	1	4	33	101	122	32	0	294
**Congenital heart anomaly**	17	14	21	34	11	8	4	0	0	109
**Endocarditis**	3	0	3	4	9	10	7	1	0	37
**Any risk-condition**	30	30	40	97	192	417	409	111	4	1330
male	15	19	20	44	111	283	257	64	2	815
female	15	11	20	53	81	134	152	47	2	515

Only patients included from the high-incidence area, stratified according to age groups and sex.

**Table 5:** Coverage of Q fever vaccination in the high-incidence area, for risk-conditions separately and combined.

Coverage and age groups	15–20	20–30	30–40	40–50	50–60	60–70	70–80	80–90	>90yrs	all	lower limit	upper limit	(all adjusted)
**Heart valve defect**	30%	14%	13%	12%	21%	16%	11%	4%	1%	11%	10%	11%	(17%)
**Aortic aneurysm / prosthesis**	0%	8%	0%	5%	12%	19%	15%	6%	0%	12%	11%	14%	(21%)
**Congenital heart anomaly**	8%	5%	14%	13%	10%	5%	10%	0%	0%	9%	7%	11%	(16%)
**Endocarditis**	10%	0%	11%	28%	13%	10%	10%	8%	0%	12%	8%	18%	(27%)
**Any risk condition**	14%	9%	13%	12%	19%	16%	13%	5%	1%	11%	11%	12%	(18%)
lower limit	8%	6%	9%	9%	15%	14%	11%	4%	1%	-	-	-	-
upper limit	24%	13%	20%	15%	23%	19%	14%	5%	1%	-	-	-	-
male	19%	10%	17%	12%	21%	20%	15%	6%	2%	14%	13%	15%	(23%)
female	11%	7%	11%	12%	16%	12%	10%	4%	1%	9%	8%	10%	(15%)

Coverage based on screened patients in the Q fever vaccination campaign from the high-incidence area and stratified for age groups and sex. Lower and upper limits based on 95%CI of the prevalence estimates. Adjusted coverage for all age groups combined calculated with the number of referred patients from the high-incidence area in the Q fever vaccination campaign.

## Discussion

This study aimed to evaluate the quantitative performance of the Q fever vaccination campaign in 2011, which targeted people at risk for chronic Q fever. Prevalence data for the defined risk-conditions were not available at the time and no disease registers exist in the Netherlands. In this retrospective cohort study, we identified the number of people with those predefined risk-conditions for chronic Q fever in a random sample of a population-based database, from which we calculated stratified prevalence estimates. By extrapolation of these prevalence data to the population of the high-incidence area of the Q fever epidemic, we arrived at an approximation of about 12,000 people with a definite or probable cardiovascular condition predisposing to chronic Q fever for this particular area. Using the recorded number of people actually screened for Q fever vaccination from this area, we estimated the overall coverage to be 11% (upper limit 12%). The adjusted coverage estimate, based on the larger group of referred people, was 18%.

There are several possible explanations for this limited coverage. First, the centralized approach, with only one location and limited dates for screening and vaccination may have posed a barrier for eligible people. Moreover, potential vaccinees had to travel twice because of the required and invasive pre-vaccination skin test and serology. As the vaccine was unregistered, information leaflets and letters were sent to cardiologists and GPs and to potential vaccinees, in which uncertainties were described explicitly; this may have influenced their willingness to refer or to be vaccinated. Furthermore, potential vaccinees needed to sign multiple consent forms, which may have been another barrier to refer or to consent. The necessity to fill in several diaries to record experienced adverse events and the intensified safety surveillance with follow-up by telephone may have put off quite some referred people. Reasons for secondary withdrawal were, among others, too far- unsafe/perceived risk benefit misbalance- intercurrent illness- feeling to be a guinea pig- difficult/impossible day/date- lack of transport- etcetera. Some potential vaccinees could not walk, were bedridden, or felt they were not at risk because they hardly ever left their home. Some other people were excluded because one felt they were unable to comply with the requirements of informed consent and follow-up. Hence the low coverage in the oldest age groups. Moreover, when the vaccination campaign finally took off, the outbreak was already in decline due to the effects of consecutive veterinary interventions in the year before; the urgency of vaccination was felt less, not only by referring GPs and their patients but also by the coordination team of the campaign and its implementers. In December 2010, HC decided not to include (new) occupational risk groups because risk of infection had sufficiently decreased [18]. This led to the decision not to extend the relative short intake period for the Q fever vaccination campaign. In contrast with population based campaigns or programmes, no repeated public education and information was launched. To avoid the so-called “worried well” phenomenon, the public were not addressed directly. The short time span between informing the public and professionals, recruitment of potential vaccinees and the screening and vaccination period, posed an obstacle to timely referral and intake. As reported [11], GPs had varying degrees of difficulty to identify eligible patients from the files depending on their file system specifications and codes. Lastly, the lack of prevalence data may have negatively influenced coverage in an indirect way, as inadequate logistics for coping with a much larger than expected number of indicated patients may have put off some patients, because convenient appointments could not easily be made.

For coverage estimation, we left out all cases labelled as possible from the IPCI-study population because many were early cases and of the mildest severity, without full clinical work up. GPs might not interpret most of these patients to be at high-risk for chronic Q fever; neither would these people think so themselves. We calculated coverage for people included in pre-vaccination screening, as this group was effectively enrolled, instead of for people referred as the larger group that was potentially reached. For this latter group, adjusted coverage would be approximately 50% higher. Coverage estimation was based on the Q fever high-incidence area (75% of all screened people), for which we had retrieved background population numbers.

The coverage estimates varied somewhat among risk-conditions and age groups. The low coverage in the two oldest age decades is not surprising in the light of the obstacles and criteria mentioned before. The highest coverage was in the (largest) middle age groups, with 19% and 16% for 50–60 and 60–70 years old, respectively. Men had higher coverage rates than women. In the light of the higher Q fever risk in these specific age groups and in men [19], this is a positive finding. These risk factors were included in the HC-advice [6] and in information letters to GPs, possibly leading to increased referral. Women were underrepresented in referral and screening for Q fever-vaccination. An explanation might be that they have milder (asymptomatic) cardiovascular defects or were less receptive to be referred for vaccination [20].

In contrast to the well-incorporated Dutch universal childhood vaccination programme (over 95% coverage, except for HPV due to concerns about safety and necessity) [21][, coverage for other (adult) risk-group vaccination is often disappointingly low, even after official and repeated recommendations [22–26]. The annual influenza vaccination programme sticks out comparatively positively but for the hepatitis B vaccination for men having sex with men the estimated annual coverage of only 1% [27]. Such risk-group targeted vaccinations are also hard to monitor, especially if executed in the private sector without disease databases and vaccination registers [28].

The calculated prevalence estimates for the four risk-conditions showed that overall, 3.1% of the population of 15 years and older had one or more of the defined conditions, increasing with age and similar for men and women. One might think that prevalence estimates could have been retrieved from literature before the start of the vaccination campaign. This could have prevented some of the logistic pressures because of the unexpected large influx of referrals. Data on prevalence of valvular defects [29–34], aortic aneurysms [35–39], congenital heart anomalies [40–45], and endocarditis [46,47] can indeed be found in literature [Table 3]. Some publications are quite recent, however, and very few are from the Netherlands [37]. There is no reason though, to expect substantial differences in prevalence rates between developed countries, but some racial or ethnic differences have been described [48], as well as changes over time, better therapeutic procedures and higher life expectancy [43].

The prevalence estimates in this study are the first available in the Netherlands. Heart valve defects, as the most common risk-condition presented in 2.4%, with peak prevalence of 17.6% in the older age groups. Aortic aneurysm/prosthesis occurred in 0.6%, with peak prevalence of 3%. Congenital cardiovascular anomalies were a rather small group in the IPCI-study population as well as in the Q fever vaccination campaign and were comparatively overrepresented in the youngest age group. This may be because patients with the more severe defects may not have survived, or recent severe degenerative or acquired symptomatic changes may have overshadowed the primary condition. Prevalence estimates for all risk-conditions seem to be well in line with the rather scarce reports in literature.

Observed prevalence rates indicate that a very large group of people in the population is at substantial risk of Q fever complications. Not only those living in the high-incidence area at the time, but also those living in the proximity of infected farms in other areas. Of note, environmental *Coxiella burnetii* has the potential to spread over great distances [49,50]. Seroprevalence studies conducted in 2009–2011 have found a large proportion (>10%) of people infected by *C*. *burnetii* and illustrates the magnitude of the Dutch Q fever outbreak [51]. For the future, containing Q fever is of great importance, and relaxation of veterinary measures, i.e. mandatory vaccination and tank milk surveillance for Q fever at goat-farms, could pose a great hazard for this prevalent risk group. Therefore, veterinary measures containing Q fever need to be continued.

A difficulty for this study was that the risk-conditions searched for in the IPCI- database required long string search terms in addition to ICPC-codes and manual validation. This was necessary because much information on these risk-conditions was captured in unstructured text in GP information systems. Medical expertise was necessary to validate the patients, because patient-records had to be assessed in medical perspective. With searches based on ICPC-codes alone, a large proportion of qualifying patients would have been missed. Patients with only an ICPC-code for risk-condition, qualified for the lowest level of diagnostic certainty (possible) at most, because free text substantiation was missing. In addition, some patients with an incorrect code would have been included without the free text validation. Some miscoding may occur after several consecutive changes in the coding system and because of misunderstanding by the coding-assistant. For instance, several times, the rheumatic fever code-in Dutch acute rheuma(tic disease)- was mistakenly chosen in cases of acute presentation of polyarthritis rheumatica. Perusing the free text entries, this could be sorted out in the majority of cases. We may have missed less obvious misconceptions, however. In addition, not all risk-conditions or targeted interventions had codes that were specific enough.

As the Q fever vaccination campaign was not designed as a study, indications for referral, especially in people with multiple risk-conditions, may not have been completely reported. On the other hand, checking of people’s eligibility was strict and all underlying risk-conditions were critically reviewed, before acceptation for pre-vaccination screening. Even so, only a small percentage (2.9%) of referred people was rejected because proper indication lacked or because of young age.

A strength of this study is that the IPCI-study population—which we used to calculate prevalence estimates of the risk-conditions—was representative of the Dutch population. The existence of population registers enabled us to extrapolate these prevalence estimates to the Q fever high-incidence area. The consistency of the yield with prevalence data available from literature and the similar relative frequencies for the respective risk-conditions in the Q fever vaccination campaign and the IPCI-study population inspire confidence. We assume high sensitivity of our search strategy with the broad search terms we chose (including spelling errors), while we did not come across other terms or synonyms for the conditions we were after in the free text of the validated records. In this perspective, the relative high proportion of rejections after validation was reassuring. We increased specificity through this individual validation of the full extracted patient group and subsequent review of all borderline cases with subsequent inclusion or rejection. Moreover, the called for risk-conditions had a relative high frequency in the general population resulting in precise estimates with small 95% confidence intervals.

In conclusion, the estimated coverage of the Q fever vaccination campaign of 11–18% was limited, but is understandable if all obstacles are taken into account. The IPCI-database extraction for determination of risk-condition prevalence rates turned out to be a feasible procedure within a limited time frame. It is fit for future use in case background rates are necessary for policymaking, implementation and surveillance of (public) health interventions, provided proper and specific (low level) codes exist for disease (sub)groups. This may be particularly true for the planned efforts to launch recommendations on the benefit of vaccinations for subgroups in addition to universal vaccination programmes [52–54]. This holds even truer for those risk-conditions for which prevalence data are missing, and the size of the targeted subgroup needs to be estimated.

## Supporting Information

S1 Table Risk-conditions for chronic Q fever in the IPCI-study population, according to age groups for cases with definite of probable diagnostic certainty. Distribution of the full IPCI-study population; number of risk-condition cases in the IPCI-study population; prevalence estimates of risk-conditions per 10,000 according to sex, as calculated from the IPCI-study population, with lower and upper limits.
(DOCX)

S2 Table Estimated numbers of people with risk-conditions for chronic Q fever in the high-incidence area. Numbers shown per age group and sex based on the prevalence rates for cases with definite and probable diagnostic certainty from the IPCI-study population.
(DOCX)
