# Supplementary material for: Coverage of the 2011 Q Fever Vaccination Campaign in the Netherlands, Using Retrospective Population-Based Prevalence Estimation of Cardiovascular Risk-Conditions for Chronic Q Fever
Source: PLoS One. 2015 Apr 24;10(4):e0123570. doi: 10.1371/journal.pone.0123570 (PMC4409345; doi:10.1371/journal.pone.0123570)
Supplement: S1 Table — Distribution of the full IPCI-study population; number of risk-condition cases in the IPCI-study population; prevalence estimates of risk-conditions per 10,000 according to sex, as calculated from the IPCI-study population, with lower and upper limits. (DOCX) [file pone.0123570.s001.docx]

**Table S1.** **Risk-conditions for chronic Q fever in the IPCI-study population, according to age groups for cases with definite of probable diagnostic certainty.** Distribution of the full IPCI-study population; number of risk-condition cases in the IPCI-study population; prevalence estimates of risk-conditions per 10,000 according to sex, as calculated from the IPCI-study population, with lower and upper limits.

|  | | | **15-20** | | **20-30** | **30-40** | **40-50** | **50-60** | **60-70** | **70-80** | **80-90** | **> 90 yrs** | **all** |
| --- | --- | --- | --- | --- | --- | --- | --- | --- | --- | --- | --- | --- | --- |
| **IPCI-study population (number)** | | |  | | | | | | | | | | |
| **all** | | | 2158 | | 4708 | 5001 | 6224 | 5509 | 4454 | 2837 | 1447 | 233 | 32571 |
|  | **male** | | 1093 | | 2354 | 2400 | 3047 | 2728 | 2138 | 1300 | 579 | 72 | 15711 |
|  | **female** | | 1065 | | 2354 | 2601 | 3177 | 2781 | 2316 | 1537 | 868 | 161 | 16860 |
|  | | **male%** | 51% | | 50% | 48% | 49% | 50% | 48% | 46% | 40% | 31% | 48% |
| **Risk-conditions (number def+prob)** | | |  | |  |  |  |  |  |  |  |  |  |
| **heart valve defect** | | | 3 | | 8 | 11 | 35 | 49 | 134 | 193 | 159 | 38 | 630 |
| **aortic aneurysm/prosthesis** | | | 0 | | 1 | 0 | 6 | 19 | 37 | 61 | 45 | 4 | 173 |
| **congenital heart anomaly** | | | 14 | | 20 | 11 | 18 | 8 | 11 | 3 | 3 | 0 | 88 |
| **endocarditis** | | | 0 | | 1 | 2 | 1 | 5 | 7 | 5 | 1 | 0 | 22 |
| **any risk condition** | | | 14 | | 26 | 22 | 57 | 72 | 176 | 236 | 198 | 40 | 841 |
|  | **male** | | 5 | | 14 | 8 | 26 | 36 | 95 | 121 | 96 | 12 | 413 |
|  | **female** | | 9 | | 12 | 14 | 31 | 36 | 81 | 115 | 102 | 28 | 428 |
| **Prevalence of risk-conditions (def+prob) (/10,000)** | | | |  | | | | | | | | | |
| **heart valve defect** | | | 14 | | 17 | 22 | 56 | 89 | 301 | 680 | 1099 | 1631 | 193 |
|  | **male** | | 9 | | 21 | 17 | 46 | 77 | 318 | 662 | 1071 | 1528 | 173 |
|  | **female** | | 19 | | 13 | 27 | 66 | 101 | 285 | 696 | 1118 | 1677 | 212 |
| **aorta aneurysm/prosthesis** | | | 0 | | 2 | 0 | 10 | 34 | 83 | 215 | 311 | 172 | 53 |
|  | **male** | | 0 | | 4 | 0 | 13 | 51 | 126 | 377 | 639 | 417 | 86 |
|  | **female** | | 0 | | 0 | 0 | 6 | 18 | 43 | 78 | 92 | 62 | 23 |
| **congenital heart anomaly** | | | 65 | | 42 | 22 | 29 | 15 | 25 | 11 | 21 | 0 | 27 |
|  | **male** | | 46 | | 42 | 17 | 30 | 15 | 19 | 15 | 17 | 0 | 25 |
|  | **female** | | 85 | | 42 | 27 | 28 | 14 | 30 | 7 | 23 | 0 | 29 |
| **endocarditis** | | | 0 | | 2 | 4 | 2 | 9 | 16 | 18 | 7 | 0 | 7 |
|  | **male** | | 0 | | 4 | 0 | 0 | 11 | 19 | 15 | 17 | 0 | 7 |
|  | **female** | | 0 | | 0 | 8 | 3 | 7 | 13 | 20 | 0 | 0 | 7 |
| **any risk-condition** | | | 65 | | 55 | 44 | 92 | 131 | 395 | 832 | 1368 | 1717 | 258 |
|  | **lower limit** | | 37 | | 38 | 29 | 71 | 104 | 342 | 736 | 1201 | 1287 | 242 |
|  | **upper limit** | | 109 | | 81 | 67 | 119 | 164 | 456 | 939 | 1555 | 2253 | 276 |
|  | **male** | | 46 | | 59 | 33 | 85 | 132 | 444 | 931 | 1658 | 1667 | 263 |
|  | **female** | | 85 | | 51 | 54 | 98 | 129 | 350 | 748 | 1175 | 1739 | 254 |
