# Supplementary material for: Coverage of the 2011 Q Fever Vaccination Campaign in the Netherlands, Using Retrospective Population-Based Prevalence Estimation of Cardiovascular Risk-Conditions for Chronic Q Fever
Source: PLoS One. 2015 Apr 24;10(4):e0123570. doi: 10.1371/journal.pone.0123570 (PMC4409345; doi:10.1371/journal.pone.0123570)
Supplement: S2 Table — Numbers shown per age group and sex based on the prevalence rates for cases with definite and probable diagnostic certainty from the IPCI-study population. (DOCX) [file pone.0123570.s002.docx]

**Table S2.** **Estimated numbers of people with risk-conditions for chronic Q fever in the high-incidence area.** Numbers shown per age group and sex based on the prevalence rates for cases with definite and probable diagnostic certainty from the IPCI-study population.

| **Eligible people high-incidence area**  **(estimated numbers)** | | 15-20 | 20-30 | 30-40 | 40-50 | 50-60 | 60-70 | 70-80 | 80-90 | >90yrs | **all** |
| --- | --- | --- | --- | --- | --- | --- | --- | --- | --- | --- | --- |
| Heart valve defect | | 47 | 102 | 152 | 498 | 703 | 1940 | 2662 | 1921 | 392 | 8782 |
|  | Lower limit | 16 | 52 | 85 | 358 | 532 | 1642 | 2322 | 1657 | 292 | 8128 |
|  | Upper limit | 138 | 202 | 271 | 691 | 928 | 2291 | 3048 | 2220 | 519 | 9490 |
| Aorta aneurysm/prosthesis | | 30^a^ | 13 | 27^a^ | 85 | 273 | 536 | 841 | 544 | 41 | 2412 |
|  | Lower limit | 0 | 2 | 0 | 39 | 175 | 389 | 657 | 408 | 16 | 2079 |
|  | Upper limit | 60 | 72 | 53 | 186 | 425 | 737 | 1077 | 723 | 104 | 2797 |
| Congenital heart anomaly | | 219 | 256 | 152 | 256 | 115 | 159 | 41 | 36 | 24^a^ | 1227 |
|  | Lower limit | 131 | 166 | 85 | 162 | 58 | 89 | 14 | 12 | 0 | 996 |
|  | Upper limit | 367 | 395 | 271 | 404 | 226 | 285 | 121 | 106 | 47 | 1511 |
| Endocarditis | | 30^a^ | 13 | 28 | 14 | 72 | 101 | 69 | 12 | 24^a^ | 307 |
|  | Lower limit | 0 | 2 | 8 | 3 | 31 | 49 | 29 | 2 | 0 | 203 |
|  | Upper limit | 60 | 72 | 100 | 81 | 168 | 209 | 161 | 68 | 47 | 464 |
| Any risk condition | | 219 | 332 | 303 | 811 | 1033 | 2549 | 3255 | 2392 | 413 | 11724 |
|  | Lower limit | 125 | 227 | 200 | 626 | 821 | 2204 | 2879 | 2100 | 310 | 10965 |
|  | Upper limit | 367 | 486 | 459 | 1049 | 1299 | 2944 | 3675 | 2719 | 542 | 12532 |
| Male | | 80 | 184 | 116 | 381 | 528 | 1446 | 1688 | 1043 | 89 | 5922 |
|  | Lower limit | 34 | 110 | 58 | 261 | 382 | 1188 | 1423 | 866 | 52 | 5384 |
|  | Upper limit | 186 | 309 | 228 | 557 | 728 | 1758 | 1996 | 1247 | 143 | 6513 |
| Female | | 138 | 149 | 184 | 428 | 505 | 1117 | 1571 | 1315 | 326 | 5808 |
|  | Lower limit | 73 | 85 | 110 | 302 | 366 | 902 | 1317 | 1094 | 231 | 5289 |
|  | Upper limit | 261 | 260 | 309 | 606 | 698 | 1382 | 1870 | 1574 | 450 | 6376 |

^a^ These numbers are estimates because no patients were found in the IPCI-sample while the screened patient group from the high-incidence area did have some.
